# PCGAN: a generative approach for protein complex identification from protein interaction networks

**DOI:** 10.1093/bioinformatics/btad473

**Published:** 2023-08-02

**Authors:** Yuliang Pan, Yang Wang, Jihong Guan, Shuigeng Zhou

**Affiliations:** Department of Computer Science and Technology, Tongji University, Shanghai 201804, China; Department of Computer Science and Technology, Tongji University, Shanghai 201804, China; Department of Computer Science and Technology, Tongji University, Shanghai 201804, China; Shanghai Key Laboratory of Intelligent Information Processing, and School of Computer Science, Fudan University, Shanghai 200438, China

## Abstract

**Motivation:**

Protein complexes are groups of polypeptide chains linked by non-covalent protein–protein interactions, which play important roles in biological systems and perform numerous functions, including DNA transcription, mRNA translation, and signal transduction. In the past decade, a number of computational methods have been developed to identify protein complexes from protein interaction networks by mining dense subnetworks or subgraphs.

**Results:**

In this article, different from the existing works, we propose a novel approach for this task based on generative adversarial networks, which is called **PCGAN**, meaning identifying **P**rotein **C**omplexes by **GAN**. With the help of some real complexes as training samples, our method can learn a model to generate new complexes from a protein interaction network. To effectively support model training and testing, we construct two more comprehensive and reliable protein interaction networks and a larger gold standard complex set by merging existing ones of the same organism (including human and yeast). Extensive comparison studies indicate that our method is superior to existing protein complex identification methods in terms of various performance metrics. Furthermore, functional enrichment analysis shows that the identified complexes are of high biological significance, which indicates that these generated protein complexes are very possibly real complexes.

**Availability and implementation:**

https://github.com/yul-pan/PCGAN.

## 1 Introduction

Protein complexes (PCs) are composed of interacting proteins, which play important roles in various cell functions, including signal transduction, protein degradation, and mRNA translation (Alberts 1998, Hartwell *et al.* 1999). With the development of high-throughput techniques, such as yeast 2-hybrid (Y2H) (Fields and Song 1989) assays and affinity purification–mass spectrometry (AP-MS) (Morris *et al.* 2014), it is cheap and fast to acquire large amounts of protein–protein interactions (PPIs) of different organisms. This provides an opportunity to identify PCs from protein interaction networks (PINs) by computational methods, which are of lower cost and higher speed, compared to biological experiments (e.g. tandem affinity purification with mass spectrometry) (Xu and Guan 2014, Shi *et al.* 2018, Yao *et al.* 2020b). A PIN can be represented as an undirected graph G=(V,E) where *V* indicates the set of proteins (or nodes) and *E* is the set of interactions (or edges). A comprehensive PIN containing more proteins and interactions is beneficial to accurately and completely identify complexes.

In the past decade, a number of computational methods have been proposed to identify PCs from PINs (Jiang and Singh 2010, Kenley and Cho 2011, Nepusz *et al.* 2012, Wu *et al.* 2021b, Pan *et al.* 2023). Most of them employ clustering algorithms by treating PCs as dense subnetworks or subgraphs in PINs (Kenley and Cho 2011, Nepusz *et al.* 2012, Wu *et al.* 2021b). Thus, the quality of PINs is crucial to the performance of complex identification. Though more and more PPI data are available, which benefits the construction of larger PINs, the overlap between different PINs of the same organism produced by different labs are quite low, especially for interactions or edges. So, it is reasonable to merge multiple PINs to construct larger and more comprehensive PINs for boosting PC identification.

In this work, we propose a novel approach PCGAN to identify PCs from PINs. It is a generative approach, which is quite different from the existing works that are mainly based on clustering analysis over PINs. Here, PCGAN means identifying **P**rotein **C**omplexes by **G**enerative **A**dversarial **N**etworks (GANs). With the help of some known complexes as training samples, PCGAN first learns the characteristics of PCs, and then generates new complexes. Specifically, PCGAN trains two models: a generator for generating PCs, and a discriminator for distinguishing the generated PCs from real ones. The competition learning between the generator and the discriminator promotes the two models to improve their capabilities until the generated complexes are indistinguishable from the real ones. Furthermore, to improve the performance of complex identification, we construct two more comprehensive PINs and a larger gold standard complex (GC) dataset by merging the existing datasets.

We conduct extensive experiments to evaluate the proposed method. Our experimental results indicate that PCGAN is superior to the existing methods. We also perform function enrichment analysis on the generated complexes, which shows that our generated PCs are of high biological significance, i.e. they are very possibly real complexes.

To the best of our knowledge, this is the first generative work of complex identification. Our method pioneers a new direction of PC identification.

## 2 Materials and methods

### 2.1 Datasets

The datasets used in this article consist of two parts: PINs and gold standard PC sets, of two organisms (human and yeast). The detailed description of the datasets is as follows:

#### 2.1.1 Protein interaction networks

For human, we used two recently released PIN datasets HuRI (Luck *et al.* 2020) and BioPlex (Huttlin *et al.* 2021), whose PINs cover a large number of human proteins and PPIs. Among them, HuRI was derived from Y2H, comprising 63 132 PPIs across 8975 proteins, and BioPlex was generated by AP-MS, consisting of 167 932 PPIs across 14 484 proteins. We merged the above two PINs and removed redundant interactions to get a more comprehensive PIN of human called **CPIN-H**, which contains 225 642 PPIs over 16 600 human proteins. As for yeast, we used five widely used PINs, including Collins (Collins *et al.* 2007), Gavin (Gavin *et al.* 2006), Krogan (Krogan *et al.* 2006), WIPHI (Kiemer *et al.* 2007), and DIP (Xenarios *et al.* 2002), which contain high reliable PPIs. Similar to human PINs, we merged the five PINs to get a more comprehensive PIN of yeast, named **CPIN-Y**. The information of PINs of human and yeast is summarized in Table 1. In this article, we used only **CPIN-H** and **CPIN-Y** for complex identification.

**Table 1.** The summary of PINs.

Organism	PIN	#Proteins	#PPIs
Human	HuRI	8975	63 132
	BioPlex	14 484	167 932
	**CPIN-H**	**16 600**	**225 642**
Yeast	Collins	1622	9074
	Gavin	1855	7669
	Krogan	2674	7079
	WIPHI	5955	50 000
	DIP	4928	17 201
	**CPIN-Y**	**6094**	**63 606**

#### 2.1.2 Gold standard PC sets

We downloaded the latest version CORUM 3.0 (Giurgiu *et al.* 2019) as the GC set of human, which contains 2485 PCs of size ≥2. For yeast, three PC datasets have been used as the gold standard sets, including CYC2008 (Pu *et al.* 2009), the Munich Information Centre for Protein Sequences (MIPS) dataset (Mewes *et al.* 2006), and the Saccharomyces Genome Database (SGD) (Hong *et al.* 2008). Here, we merged the three datasets and removed the redundant complexes (if two complexes exactly match each other, they are redundant to each other) to obtain a larger gold standard set of yeast (named **CGold-Y**). Table 2 provides the statistics of these gold standard PC sets. In experiments, we used only CORUM and **CGold-Y**.

**Table 2.** The summary of gold standard PC sets.

Organism	Dataset	#Proteins	#PCs
Human	CORUM	3382	2485
Yeast	CYC2008	1627	349
	MIPS	1228	273
	SGD	1272	306
	**CGold-Y**	**1984**	**711**

### 2.2 Methodology

**Figure 1.**
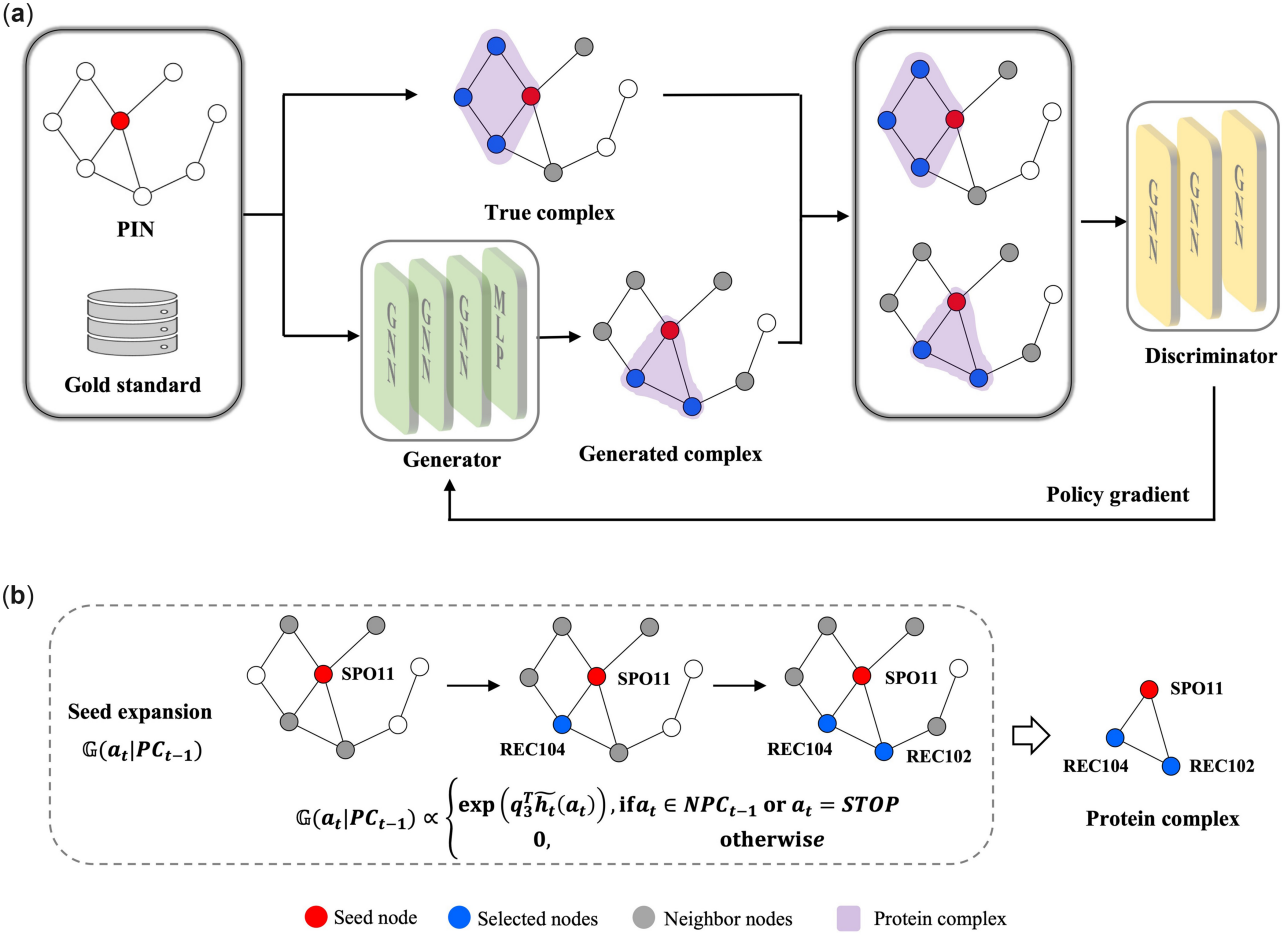
The architecture and complex generation process of PAGAN. (a) The architecture of PCGAN. The input data of PCGAN includes a PIN and a gold standard dataset. The generator is to generate complexes as similar as possible to true complexes. The discriminator tries to distinguish between true and generated complexes, and returns gradient updates to make the generator generate high-quality complexes. The iterative learning process improves the generator and the discriminator until generated complexes are indistinguishable from true complexes. (b) The illustration of complex generation process. A complex is generated by starting from a seed node. The policy $G\left(a_{t}|PC_{t\;-\;1}\right)$ models the complex generation by iteratively expanding the current generated intermediate complex. Some examples of failed and successful complexes generated by PCGAN are presented in the Supplementary Material (see Supplementary Fig. S1).

Here, we first introduce the framework of PCGAN as shown in Fig. 1, then present the techniques of the discriminator and generator in PCGAN. Finally, we describe the algorithm.

#### 2.2.1 The framework of PCGAN

Figure 1a shows the architecture of PCGAN based on GANs, where the generator is to generate PCs, each of which is started with a seed node, and expanded iteratively based on the policy gradient strategy. The discriminator is to distinguish a generated PC from real ones. The competition learning between the generator and the discriminator drives them to improve their capabilities until the generated complexes are indistinguishable from the true complexes. In detail, the input data of PCGAN include a PIN (CPIN-H or CPIN-Y) and a GC dataset (CORUM or CGold-Y). *N* complexes are selected from the GC dataset to form the training set, and the rest are used as the test set. Among them, 30% of the training set is for model verification, i.e. evaluating the quality of the model in the training process. To generate a complex, a node is randomly selected from the PIN as the seed node, and input to the generator. In the process of complex generation, an optimal node from the neighbors of the current (intermediate) complex is chosen to join the current complex or terminate the expansion of the current (intermediate) complex. As each node in the PIN will be selected for complex generation, the order will not impact the final result. Next, the true complexes and the generated complexes are input into the discriminator, which returns the gradient update to the generator, to make it generate more high-quality complexes. Finally, the proteins of complexes in the test set are input into the trained generator to generate candidate PCs. And the final PC set is obtained by removing redundant complexes according to the complex match rate $\mathrm{Rat}e_{\mathrm{match}}$ [Equation (10)]. As an example, Fig. 1b illustrates the generation process of a real complex. In this article, we use the notation in Table 3. In addition, lowercase letters (e.g. *x*) represent column vectors, and uppercase letters (e.g. *X*) denote sets.

**Table 3.** The notation of this article.

Symbol	Description
G=(V,E)	A PIN or graph
PC	A protein complex
$PC_{t}=\left\{v_{0},\ldots ,v_{t}\right\}$	An intermediate complex at step *t*
NPC	The set of neighbor nodes of a protein complex
C=PC+NPC	A protein complex with its neighbor nodes
D, G	Discriminator and generator

#### 2.2.2 Discriminator

A PIN can be represented by an undirected graph G=(V,E), where *V* is a set of vertices (i.e. proteins) and *E* is the set of edges (i.e. interactions). The discriminator D aims to judge whether a PC is real or generated. Here, we use a graph isomorphism network (GIN) (Welling and Kipf 2016, Gasteiger *et al.* 2018, Xu *et al.* 2018) to construct the discriminator of PCGAN because it is effective in representing graphs and shows good performance in graph classification. To characterize the features of complexes in the PIN, we consider both internal and external connections of a PC, i.e. C=PC+NPC, where *PC* represents the internal node set of the PC, and *NPC* represents its neighbor node set.

The discriminator is a “three”-layer GIN, and the initial features of nodes are evaluated as follows: where *v* is a node in the PIN, and $w_{0}$, $w_{1}$, and $w_{2}\in R^{d}$ are parameters. ℓ is the condition function, which returns 1 when the condition is true, otherwise 0. At layer l (l≤3), the representation of node *v* is evaluated as follows: above, $m^{l}(v)$ and $z^{l}(v)$ are intermediate representation vectors of node *v*, and σ is the activation function. $N_{C}(v)$ represents the neighbor node set of *v* in *C*, and $w_{l}$ is the weight vector at the *l*-th layer. The final representation of *v* is obtained by concatenating the representations of each layer: $z(v)=\left[z^{0}(v),\ldots ,z^{3}(v)\right]$. Then, we aggregate the representations of nodes in *C* by the readout function to generate the representation of *C*: $z(C)=\sum_{v\in C}\;z(v)$. Finally, we use the sigmoid function to obtain the probability that the PC is a real complex: $D(C;W)={\left[1+\text{exp}(w_{3}^{T}z(C))\right]}^{-1}$, where *W* means the parameter set of D, and $w_{3}\in W$.


(1) $$z^{0}(v)=w_{0}+\ell \left\{v\in PC\right\}\cdot w_{1}+\ell \left\{v\in \mathrm{NPC}\right\}\cdot w_{2},$$

(2) $$m^{l}(v)=\sigma \left(\sum_{u\in N_{C}(v)}z^{l-1}(u)\right),$$

(3) $$z^{l}(v)=w_{l}(z^{l-1}(v)+m^{l}(v)),$$

#### 2.2.3 Generator

Given a seed node $v_{0}$, the generator G tries to generate an optimal PC (that has the feature of high cohesion and low coupling) containing node $v_{0}$: $G(v_{0})=PC=\left\{v_{0},v_{1},\ldots ,v_{T}\right\}$ in an iterative way. Specifically, given the current intermediate complex $PC_{t-1}=\left\{v_{0},v_{1},\ldots ,v_{t-1}\right\}$, we select a node $v_{t}$ from $NPC_{t-1}$ to expand $PC_{t-1}$ or stop the expansion of the complex at the *t*-th step (t≤T). *T* represents the number of proteins or nodes contained in the final PC. Here, we use the policy $G\left(a_{t}|PC_{t\;-\;1}\right)$ to model the expansion of the current complex. $a_{t}$ represents the next action, i.e. adding a node to the current complex or stopping the expansion process. As an example, the complex expansion process is illustrated in Fig. 1b.

1) The design of policy $G\left(a_{t}|PC_{t\;-\;1}\right)$.
  At the *t*-th step, the augmented initial feature of node *v* is as follows:
  (4) $$h_{t}^{0}(v)=q_{0}+\ell \left\{v=v_{0}\right\}\cdot q_{1}+\ell \left\{v\in PC_{t-1}\right\}\cdot q_{2},$$
  where $q_{0}$, $q_{1}$, and $q_{2}\in R^{d}$ are parameters. This indicates that the feature of node *v* contains the information of the current complex and the seed node. Then, the node feature is processed by a graph neural network (GNN) model. Here, we use the Graph Pointer Network with incremental updates (iGPN) model (Zhang *et al.* 2020), which consists of three layers of GNN and a multi-layer perception (Longstaff and Cross 1987), to process node features as follows:
  (5) $${\tilde{H}}_{t}(C_{t-1})=\mathrm{iGPN}(H_{t}^{0}(C_{t-1});Q),$$
  where ${\tilde{H}}_{t}(C_{t-1})$ and $H_{t}^{0}(C_{t-1})$ are stacked node representations of $C_{t-1}$, $C_{t-1}$ denotes the set of internal and neighboring nodes of complex $PC_{t-1}$, *Q* represents the set of parameters in iGPN. iGPN is specially designed for sequential decision problems on graphs, which has the advantage of less training time and memory (Zhang *et al.* 2020).
  Then, we design the action representation ${\tilde{h}}_{t}(a_{t})$, which determines whether to add a node to the current complex $PC_{t-1}$ or to end the expansion process via a probability:
  (6) $$G\left(a_{t}|PC_{t-1}\right)\propto \{\begin{array}{cc} \;\text{exp}(q_{3}^{T}{\tilde{h}}_{t}(a_{t})), & \;\text{if}\;a_{t}\in NPC_{t-1}\;\text{or}\;a_{t}=\mathrm{STOP}\\ 0, & \text{otherwise} \end{array}.$$
  Specifically, the STOP action representation is defined as follows:
  (7) $${\tilde{h}}_{t}(\mathrm{STOP})=\frac{\sum_{v\in PC_{t-1}}^{}\alpha_{t}(v)\cdot {\tilde{h}}_{t}(v)}{\sum_{v\in PC_{t-1}}^{}\alpha_{t}(v)\cdot {\tilde{h}}_{t}(v)+\sum_{u\in NPC_{t-1}}^{}\alpha_{t}(u)\cdot {\tilde{h}}_{t}(u)},$$
  (8) $$\alpha_{t}(v\;or\;u)=\frac{\;\text{exp}(q_{4}^{T}{\tilde{h}}_{t}(v\;or\;u))}{\sum_{v\in PC_{t-1}\;\;or\;\;u\in NPC_{t-1}}\;\text{exp}\;(q_{4}^{T}{\tilde{h}}_{t}(v\;or\;u))},$$
  where $q_{3}$ and $q_{4}$ are parameters. The iGPN parameter set *Q* = $\left\{q_{0},q_{1},q_{2},q_{3},q_{4}\right\}$.
2) Optimizing the generator with policy gradient.
  Here, the generator is trained by the policy gradient strategy (Sutton *et al.* 1999). We define the reward for the intermediate PC as r(PC)=−log(1−D(PC)). The generator tries to maximize the expected reward for a given seed $v_{0}$, its policy gradient relative to *Q* is
  (9) $$\nabla J_{G}(Q|v_{0})=\nabla E_{PC|v_{0}∼G}\left[r(PC)\right]=E_{v_{1},\ldots ,v_{T}|v_{0}∼G}\left[\sum_{t=1}^{T}\nabla \;\text{log}\;G(v_{t}|PC_{t-1})\cdot S(PC_{t-1},v_{t})\right],$$
  where $S(PC_{t-1},v_{t})=E_{v_{t+1},\ldots ,v_{T}|PC_{t}∼G}\left[r(PC)\right]$ represents the state-action function. We use Monte-Carlo estimation to approximate the policy gradient as in previous studies (Yu *et al.* 2017).
3) Pre-training and teacher forcing.
  Pre-training can provide a well initial model, and teacher forcing can effectively prevent the model from deteriorating and getting stuck in some training batches with the help of supervised learning in each reinforcement learning step. Here, we use maximum log likelihood estimation (MLE) for model pre-training and do teacher forcing as described in Vinyals *et al.* (2015) and Zhang *et al.* (2020).
4) Algorithm.
  The overall PCGAN algorithm is outlined in Algorithm 1, which consists of four parts: (i**)** pre-training the generator G on the training set *Ta* using MLE (Line 1); (ii**)** iteratively training the discriminator D (Lines 3–8) and the generator G (Lines 9–14) on the training set *Ta*. (iii**)** Generating complexes one by one with the trained generator on the testing set *Te* (Lines 16–19). (iv**)** Removing duplicate complexes from the generated complexes (Lines 20–23).

Algorithm 1 PCGAN

**Require:** PIN G=(V,E), Complex training set *Ta*, Complex testing set *Te*

1: Pre-train G on *Ta* by using MLE

2: **for** epoch = 1,**… do**

3: **_ _for** D_step = 1,**… do**

4: _**  **_Sample batch $B_{\mathrm{true}}\subset Ta$

5: _**  **_Sample seeds S⊂V

6: _**  **_Generate protein complexes $B_{\mathrm{false}}=\left\{G(v)|v\in S\right\}$

7: _**  **_Train D on $B=B_{\mathrm{true}}\cup B_{\mathrm{false}}$

8: **_ _end for**

9: **_ _for** G_step = 1,**… do**

10: _**  **_Sample batch $B_{\mathrm{true}}\subset Ta$

11: _**  **_Teacher-force G on $B_{\mathrm{true}}$ by using MLE

12: _**  **_Sample seeds S⊂V

13: _**  **_Train G on S with policy gradient in Equation (9)

14: **_ _end for**

15: **end for**

16: **for** each protein *u* in *Te*

17: _** **_Generate protein complex PC=G(u)

18: _** **_Protein complex set PCs=PCs∪PC

19: **end for**

20: **for** any two complexes $PC_{i}$ and $PC_{j}$ in *PCs*

21: **_ _if**$\mathrm{Rat}e_{\mathrm{match}}(PC_{i},PC_{j})=1$**then**

22: _**  **_Remove the $PC_{j}$ from *PCs*

23: **end for**

24: **return** The final protein complex set *PCs*

### 2.3 Performance metrics

To fairly compare the performance of various methods, we use four commonly used metrics, including “Recall,” “Precision,” “*F-*measure,” and “maximum matching ratio” (MMR). Before describing these metrics, we first introduce $\mathrm{Rat}e_{\mathrm{match}}$, which is used to measure the similarity between a predicted complex (PC) and a GC. |PC∩GC| represents the number of common proteins between the predicted complex and the GC. |PC| and |GC| represent the number of proteins in the predicted complex and the GC, respectively. Following previous works (Wang *et al.* 2019, Wu *et al.* 2020), if $\mathrm{Rat}e_{\mathrm{match}}\geq 0.2$, we think PC and GC match successfully. The four performance metrics are defined as follows: above, $P_{c}$ and $G_{c}$ represent the number of complexes in the predicted set and the gold standard set, respectively. $P_{gc}$ is the number of predicted complexes matching with some GCs, and $G_{pc}$ is the number of GCs matching with some predicted complexes. “*F-*measure” is the harmonic mean of “Recall” and “Precision.”


(10) $$\mathrm{Rat}e_{\mathrm{match}}=\frac{{\left|PC\cap GC\right|}^{2}}{\left|PC\right|\times \left|GC\right|}.$$

(11) $$\mathrm{Precision}=\frac{P_{gc}}{P_{c}},$$

(12) $$\mathrm{Recall}=\frac{G_{pc}}{G_{c}},$$

(13) $$F-\mathrm{measure}=\frac{2\times \mathrm{Recall}\times \mathrm{Precision}}{\mathrm{Recall}+\mathrm{Precision}},$$

MMR (Nepusz *et al.* 2012) is calculated based on the maximum matching in the bipartite graph where the two node sets of the graph are the predicted complex set and the GC set, respectively. Then, dividing the sum of the maximum matching edge weights by the number of GCs, and the maximum matching edge weight is evaluated by $\mathrm{Rat}e_{\mathrm{match}}$.

### 2.4 Complex function enrichment

Currently, though the scales of PINs are stably growing, the GC set has not been updated correspondingly. This means that the known PCs are very limited, which is not beneficial to validate the generated complexes, while experimental validation is too expensive and time consuming. Here, we try another way to evaluate the effectiveness of PC prediction. In general, the higher the biological significance of a complex, the more likely it is a real complex (Wang *et al.* 2019, Omranian *et al.* 2021). Thus, we assess the biological significance of predicted PCs by functional enrichment analysis.

Here, g: Profiler (Raudvere *et al.* 2019), a popular method of functional enrichment analysis, is used to evaluate the *P*-value of each generated PC to measure its biological significance. g: Profiler uses multiple test corrections to obtain *P*-values from GO and pathway enrichment analysis. Given an input query size, g: Profiler analyzes the approximate threshold *t*, which corresponds to the 5% upper quantile of randomly generated queries of that size. All actual *P*-values resulting from the query are calibrated by multiplying these values with the ratio of the approximate threshold *t* over the initial experiment-wide threshold. In this study, we use a default *P*-value threshold 0.05, i.e. if the *P*-value of a complex is smaller than 0.05, it is biologically significant. All the proteins in the PIN (i.e. CPIN-H of human and CPIN-Y of yeast) are used as the background set and we ignore the annotations.

## 3 Results and analysis

### 3.1 Data integration

Here, we discuss and analyse the integration results of PINs and gold standard PCs.

#### 3.1.1 PIN integration

The quality of PINs is essential for computationally identifying PCs from PINs. Therefore, we examined the overlaps of proteins and PPIs between PINs of human and yeast in Table 1. For human, we found that the protein overlap rate is 47% (BioPlex overlapping HuRI) and 76% (HuRI overlapping BioPlex), but the overlap of PPIs is very low, 1% (BioPlex overlapping HuRI) and 2.6% (HuRI overlapping BioPlex). This indicates that PPIs are incomplete for any single PIN, which is harmful to complex identification (some complexes cannot be identified). Here, the rate of *A* overlapping *B* is evaluated by |A∩B|/|A|, *A* and *B* represent two PINs of the same organism, |•| is the number of proteins or PPIs in a certain PIN. There are three reasons for limited PPI overlapping between different PINs: (i**)** different high-throughput experiments tend to find different types of PPIs (Drew *et al.* 2021); (ii**)** a PIN is a dynamic network, PPIs change in cell at any time (Shi *et al.* 2018); (iii**)** current PPI experimental techniques may produce false-positive interactions (Yao *et al.* 2020a). Therefore, we merge the two PINs of human (i.e. HuRI and BioPlex) and remove the redundant interactions to construct a more comprehensive PIN, called CPIN-H, which covers more proteins and PPIs.

In addition, we have conducted complex identification on HuRI, BioPlex, and CPIN-H. The performance comparisons of PCGAN on the three PINs are presented in the Supplementary Material (see Supplementary Table S1). We can see that the prediction performance on the CPIN-H is better than that on HuRI and BioPlex. This shows that the combination of multiple different PINs is beneficial to PC identification.

Tables 4 and 5 present the overlap rates of proteins and PPIs in different PINs of yeast, respectively. Similarly, we can see that the overlap of proteins between different yeast PINs is very high, but the overlap of interactions is very low. Therefore, we combine the five different yeast PINs and remove the redundant interactions to generate a comprehensive yeast PIN, called CPIN-Y. Subsequent complex identification experiments are carried out on CPIN-H and CPIN-Y, respectively.

**Table 4.** Protein overlap of yeast PINs.

Dataset	Collins	Gavin	Krogan	WIPHI	DIP
Collins		0.842	0.840	0.998	0.935
Gavin	0.736		0.741	0.993	0.349
Krogan	0.509	0.514		0.993	0.889
WIPHI	0.272	0.311	0.446		0.806
DIP	0.308	0.349	0.483	0.974	

**Table 5.** PPI overlap of yeast PINs.

Dataset	Collins	Gavin	Krogan	WIPHI	DIP
Collins		0.249	0.146	0.352	0.132
Gavin	0.294		0.122	0.534	0.174
Krogan	0.188	0.133		0.272	0.109
WIPHI	0.063	0.082	0.039		0.094
DIP	0.070	0.078	0.045	0.275	

#### 3.1.2 Integrating PC sets

The GC sets are used to evaluate the performance of computational methods in identifying PCs. Therefore, a larger gold standard set will make the evaluation more accurate. We usually think that a complex is a complete graph. That is, any two proteins in the complex is interacted. For each complex in a gold standard set, we checked whether it is a full graph in a certain PIN. The results are presented in Table 6, from which we can see that only a small number of complexes in a gold standard set are fully connected in a PIN. These fully connected complexes are easy to identify by computational methods. For example, CYC2008 has 349 complexes, but only 138 complexes are fully connected in Collins. And each gold standard set contains a limited number of PCs. Therefore, we combined these three GC sets to generate a more comprehensive GC set of yeast, called CGold-Y, which contains more complexes, and the number of fully connected protein complexes (FPCs) substantially increases in each yeast PIN.

**Table 6.** The number of FPCs in each yeast PIN.^a^

Dataset	#PCs	#FPCs (Collins)	#FPCs (Gavin)	#FPCs (Krogan)	#FPCs (WIPHI)	#FPCs (DIP)
CYC2008	349	138	113	137	291	206
MIPS	273	84	74	75	175	137
SGD	306	128	96	112	230	152
**CGold-Y**	**711**	**240**		**225**	**518**	**351**

a The second column (“#PCs”) is the total number of protein complexes contained in each dataset.

### 3.2 Performance comparison with existing methods

Since PCGAN needs some real complexes to guide model learning, we use the remaining complexes (those not used as training samples) in the merged gold standard set as testing samples. Specifically, for human, we used 500 complexes in CORUM as the training set, 30% of the training set as the verification set, and the rest as the test set. For yeast, we used 300 in CGold-Y as the training set, 30% of the training set as the verification set, and the rest as the test set. All methods identify PCs from CPIN-H (for human) and CPIN-Y (for yeast), and compare the predicted complexes with the gold standard test set.

Table 7 shows the performance of different methods on CPIN-H and CPIN-Y. We compared PCGAN with various major existing methods, including Markov Clustering (MCL) (Enright *et al.* 2002), GraphEntropy (Kenley and Cho 2011), ClusterONE (Nepusz *et al.* 2012), SPICi (Jiang and Singh 2010), MCODE (Bader and Hogue 2003), Core (Leung *et al.* 2009), ProRank+ (Hanna and Zaki 2014), and DPClus (Altaf-Ul-Amin *et al.* 2006). For human, our method PCGAN shows advantageous results over other methods in most performance metrics, only slightly lower than ProRank+ (Hanna and Zaki 2014) in the metric of Precision. Although the ProRank+ has a higher precision, its recall is much lower, indicating that it correctly predicts only much less complexes, i.e. most of the complexes in its predicted set are successfully matched with only a small number of complexes in the gold standard set. More importantly, our method PCGAN performs much better than the other methods in terms of the comprehensive metrics *F*-measure and MMR, which indicates that our method is more effective in identifying PCs than the other methods. For yeast, PCGAN is superior to the other methods in terms of all metrics. In summary, the results above show that PCGAN is effective in identifying PCs, it can generate high-quality complexes from both yeast and human PINs.

**Table 7.** Performance comparison on human and yeast PINs.

	Human					Yeast				
Method	#PC	Recall	Precision	*F*-measure	MMR	#PC	Recall	Precision	*F*-measure	MMR
MCL	2373	0.205	0.093	0.128	0.040	1093	0.397	0.113	0.175	0.123
GraphEntropy	1294	0.091	0.111	0.100	0.019	448	0.056	0.049	0.052	0.017
ClusterONE	1126	0.181	0.181	0.181	0.036	1386	0.365	0.088	0.142	0.110
SPICi	2051	0.120	0.055	0.076	0.022	754	0.370	0.134	0.197	0.111
MCODE	120	0.008	0.092	0.015	0.002	106	0.049	0.151	0.074	0.022
Core	2853	0.165	0.076	0.104	0.035	1211	0.433	0.116	0.182	0.135
ProRank+	302	0.044	**0.225**	0.073	0.011	125	0.049	0.152	0.073	0.017
DPClus	2820	0.210	0.069	0.104	0.038	1069	0.504	0.131	0.208	0.171
PCGAN	1942	**0.288**	0.213	**0.245**	**0.058**	1259	**0.523**	**0.259**	**0.346**	**0.187**

Note: Since the evaluation depends on four evaluation metrics, each method uses default parameters.

### 3.3 Functional enrichment analysis

Due to the incompleteness of existing gold standard sets, which cannot validate all generated complexes. Thus, in this article, functional enrichment analysis is employed to verify the effectiveness of the proposed method. Concretely, we used g:Profiler (Raudvere *et al.* 2019) for functional enrichment analysis of generated (for our method) or predicted (for the other methods) complexes. The functional enrichment degree of a PC is measured by its *P*-value. The smaller the *P*-value of a complex is, the more significant its biological function is. The *P*-value threshold of significance is set to .05 by default. Table 8 shows the percentage of biologically significant complexes identified by different methods. Compared with the other methods, PCGAN identifies the largest percentage (54.88%) of biologically significant identified complexes on the yeast PIN. At the threshold of ≤1e-3, the proportion of significant complexes (30.98%) is only slightly lower than that of MCODE (32.08%). However, our method PCGAN identifies more PCs than MCODE. For human, the percentage of biologically significant complexes (75.18%) is less than that of ClusterONE (Nepusz *et al.* 2012), MCODE (Bader and Hogue 2003), and ProRank+ (Hanna and Zaki 2014). On the one hand, the size of the complexes identified by these methods is >2, which leads to low *P*-values. On the other hand, these three methods identify much less complexes than our method PCGAN. In summary, PCGAN can effectively generate PCs of high biological significance.

**Table 8.** Results of functional enrichment analysis on PCs identified by different methods.^a^

	Human			Yeast		
Method	#PC	#PC of extremely significance	#PC of significance	#PC	#PC of extremely significance	#PC of significance
MCL	2373	641 (27.01)	1413 (59.54)	1093	163 (14.91)	368 (33.67)
GraphEntropy	1294	252 (19.47)	691 (53.40)	488	47 (9.63)	122 (25.00)
ClusterONE	1126	595 (52.84)	877 (77.89)	1386	187 (13.49)	438 (31.60)
SPICi	2051	414 (20.19)	1200 (58.51)	754	170 (22.55)	317 (42.04)
MCODE	120	74 (61.67)	103 (85.83)	106	34 (32.08)	57 (53.77)
Core	2853	702 (24.61)	1645 (57.66)	1211	245 (20.23)	451 (37.24)
ProRank+	302	198 (65.56)	255 (84.44)	125	34 (27.20)	60 (48.00)
DPClus	2820	701 (24.86)	1687 (59.82)	1069	240 (22.45)	456 (42.66)
PCGAN	1942	485 (24.97)	1460 (75.18)	1259	390 (30.98)	691 (54.88)

a Column “#PC” is the total number of identified complexes by each method; column “Extremely significance” is the number of complexes of *P*-value≤1e-3 with its percentage in #PC in the parenthesis; column “Significance” is the number of complexes of *P*-value<.05 with its percentage in #PC in the parenthesis.

### 3.4 Case study

The value of a PC identification method lies in its capability of identifying unknown real PCs. However, even if a method does identify some new and real complexes, it is difficult to validate that they are real complexes without the help of biological experiments. But biological experiment validation is expensive and time-consuming.

**Figure 2.**
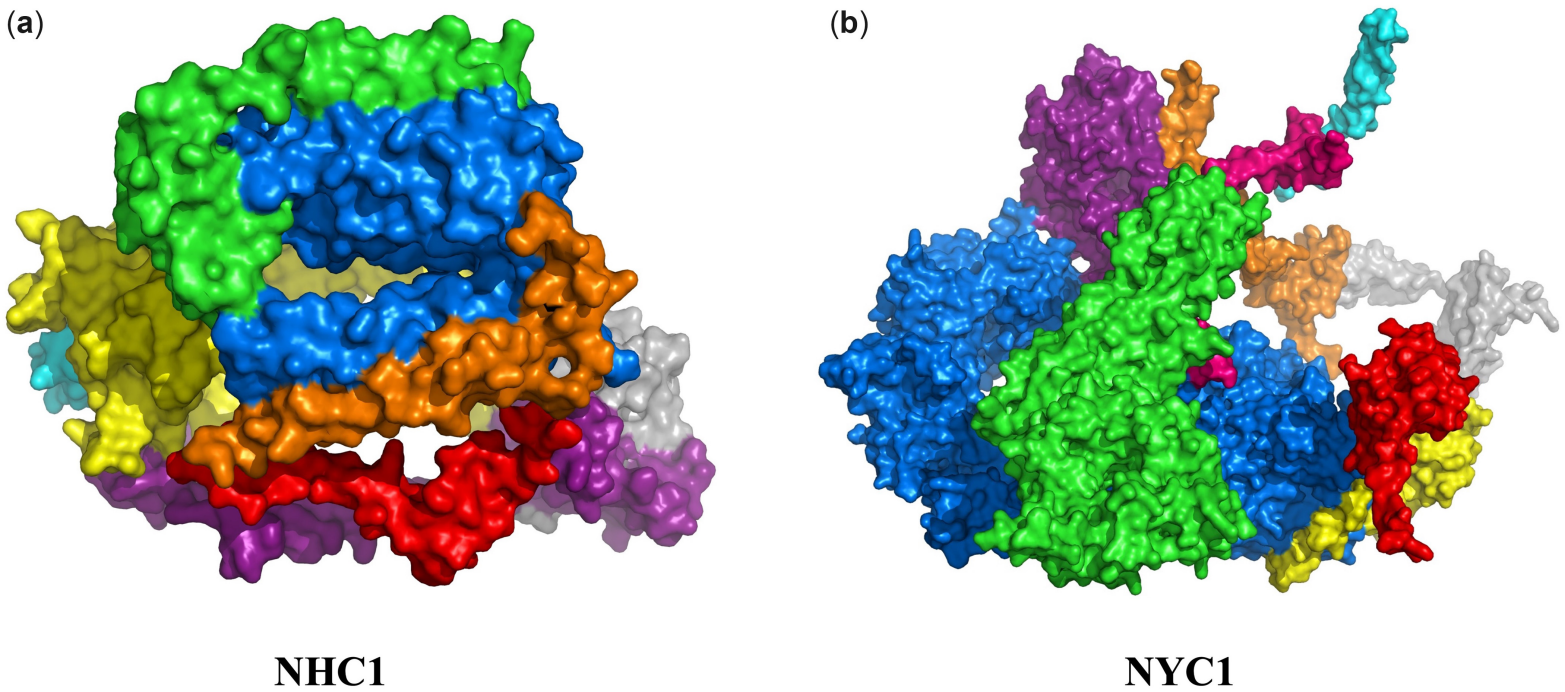
Possible real PCs generated by our method. (a) Human PC NHY1 (blue: MT-CO3, green: COX6A1, purple: COX4I1, yellow: MT-CO2, red: COX7C, gray: UQCRC2, orange: COX7A2L, and cyan: COX7A1). (b)Yeast PC NYC1 (blue: APC1, green: APC4, purple: CDC23, yellow: DOC1, red: APC11, gray: APC9, orange: SWM1, cyan: CDC26, and pink: MND2).

Here, we provide two PCs generated by our method as examples, which are very possibly real complexes according to our functional enrichment analysis and function check. These two PCs are not in the identified results of the other methods. We got the 3D structures of the two PCs by AlphaFold-Multimer (Evans *et al.* 2021), rendered the 3D structures using PyMOL (DeLano 2002). The first was generated from the human PIN, shown in Fig. 2a, we denote it by NHC1 (meaning novel human complex No. 1). NHC1 is composed of eight human proteins and its *P*-value of functional enrichment is 1.16E-17, indicating that it has high biological significance. By further function check, we found that these proteins in NHC1 mainly exist in the mitochondria of cells, involving in biological processes of aerobic respiration and oxidative phosphorylation. This implies that it may be a PC related to cell respiratory function. Another is a yeast complex shown in Fig. 2b, which is named NYC1 (meaning novel yeast complex No. 1). NYC1 consists of nine proteins, and its *P*-value of functional enrichment is 4.00E-20. We found that the significant functional enrichment of NYC1 is mainly contributed by protein ubiquitination and cell mitosis. This suggests that NYC1 may be a nuclear ubiquitin ligase complex or a complex related to cell mitosis.

## 4 Conclusion

Accurate identification of PCs from PINs is an important research topic in computational biology (Wu *et al.* 2021a). In this article, we proposed a novel approach PCGAN for identifying PCs by GANs. To the best of our knowledge, this is the first generative method for PC identification. Existing methods are mainly based on unsupervised learning—clustering over PINs by assuming that complexes are dense subnetworks of PINs. Our work provides a new framework for future research, which may inspire a new wave of studies on complex identification. Furthermore, considering the limitations of small and noisy PINs and small GC sets in previous works, we merged existing PINs of human and yeast to get larger and more comprehensive PINs, and combined existing GC sets to construct a large GC set. This not only boost the performance of our method, but also provide new sources for future research on complex identification. Our experimental results show that PCGAN outperforms the existing methods, and the generated complexes have high biological significance. As for future work, we will focus on two directions: (i**)** extending the proposed method to identify PCs from PINs with biological features; and (ii**)** designing more advanced deep generative models (e.g. diffusion models) for PC generation.

## Supplementary Material

btad473_Supplementary_Data

## Data Availability

The datasets and method code of PCGAN can be downloaded in GitHub (https://github.com/yul-pan/PCGAN).

## References

[btad473-B1] Alberts B. The cell as a collection of protein machines: preparing the next generation of molecular biologists. Cell 1998;92:291–4. 9476889 10.1016/s0092-8674(00)80922-8

[btad473-B2] Altaf-Ul-Amin M , Shinbo Y, Mihara K et al Development and implementation of an algorithm for detection of protein complexes in large interaction networks. BMC Bioinformatics 2006;7:207–13. 16613608 10.1186/1471-2105-7-207 PMC1473204

[btad473-B3] Bader GD , Hogue CW. An automated method for finding molecular complexes in large protein interaction networks. BMC Bioinformatics 2003;4:2–27. 12525261 10.1186/1471-2105-4-2 PMC149346

[btad473-B4] Collins SR , Kemmeren P, Zhao X-C et al Toward a comprehensive atlas of the physical interactome of *Saccharomyces cerevisiae*. Mol Cell Proteomics 2007;6:439–50. 17200106 10.1074/mcp.M600381-MCP200

[btad473-B5] DeLano WL PyMOL: an open-source molecular graphics tool. CCP4 Newsl Protein Crystallogr 2002;40:82–92.

[btad473-B6] Drew K , Wallingford JB, Marcotte EM et al hu.MAP 2.0: integration of over 15,000 proteomic experiments builds a global compendium of human multiprotein assemblies. Mol Syst Biol 2021;17:e10016. 33973408 10.15252/msb.202010016 PMC8111494

[btad473-B7000] Evans R et al Protein complex prediction with AlphaFoldMultimer. BioRxiv 2021:2021–10.

[btad473-B7] Enright AJ , Van Dongen S, Ouzounis CA et al An efficient algorithm for large-scale detection of protein families. Nucleic Acids Res 2002;30:1575–84. 11917018 10.1093/nar/30.7.1575 PMC101833

[btad473-B8] Fields S , Song O-K. A novel genetic system to detect protein–protein interactions. Nature 1989;340:245–6. 2547163 10.1038/340245a0

[btad473-B18] Gasteiger J , Bojchevski A, Günnemann S. Predict then propagate: graph neural networks meet personalized PageRank. arXiv, arXiv:1810.05997, 2018, preprint: not peer reviewed.

[btad473-B9] Gavin A-C , Aloy P, Grandi P et al Proteome survey reveals modularity of the yeast cell machinery. Nature 2006;440:631–6. 16429126 10.1038/nature04532

[btad473-B10] Giurgiu M , Reinhard J, Brauner B et al CORUM: the comprehensive resource of mammalian protein complexes—2019. Nucleic Acids Res 2019;47:D559–63. 30357367 10.1093/nar/gky973 PMC6323970

[btad473-B11] Hanna EM , Zaki N. Detecting protein complexes in protein interaction networks using a ranking algorithm with a refined merging procedure. BMC Bioinformatics 2014;15:204–11. 24944073 10.1186/1471-2105-15-204 PMC4230023

[btad473-B12] Hartwell LH , Hopfield JJ, Leibler S et al From molecular to modular cell biology. Nature 1999;402:C47–52. 10591225 10.1038/35011540

[btad473-B13] Hong EL , Balakrishnan R, Dong Q et al Gene ontology annotations at SGD: new data sources and annotation methods. Nucleic Acids Res 2008;36:D577–81. 17982175 10.1093/nar/gkm909 PMC2238894

[btad473-B14] Huttlin EL , Bruckner RJ, Navarrete-Perea J et al Dual proteome-scale networks reveal cell-specific remodeling of the human interactome. Cell 2021;184:3022–40.e28. 33961781 10.1016/j.cell.2021.04.011 PMC8165030

[btad473-B15] Jiang P , Singh M. SPICi: a fast clustering algorithm for large biological networks. Bioinformatics 2010;26:1105–11. 20185405 10.1093/bioinformatics/btq078 PMC2853685

[btad473-B16] Kenley EC , Cho Y-R. Detecting protein complexes and functional modules from protein interaction networks: a graph entropy approach. Proteomics 2011;11:3835–44.

[btad473-B17] Kiemer L , Costa S, Ueffing M et al WI-PHI: a weighted yeast interactome enriched for direct physical interactions. Proteomics 2007;7:932–43. 17285561 10.1002/pmic.200600448

[btad473-B19] Krogan NJ , Cagney G, Yu H et al Global landscape of protein complexes in the yeast *Saccharomyces cerevisiae*. Nature 2006;440:637–43. 16554755 10.1038/nature04670

[btad473-B20] Leung HCM , Xiang Q, Yiu SM et al Predicting protein complexes from PPI data: a core-attachment approach. J Comput Biol 2009;16:133–44. 19193141 10.1089/cmb.2008.01TT

[btad473-B21] Evans R , O’Neill M, Pritzel A, et al Protein complex prediction with AlphaFold-Multimer. *biorxiv*, 2021.

[btad473-B22] Longstaff ID , Cross JF. A pattern recognition approach to understanding the multi-layer perception. Pattern Recognit Lett 1987;5:315–9.

[btad473-B23] Luck K , Kim D-K, Lambourne L et al A reference map of the human binary protein interactome. Nature 2020;580:402–8. 32296183 10.1038/s41586-020-2188-x PMC7169983

[btad473-B24] Mewes HW , Frishman D, Mayer KFX et al MIPS: analysis and annotation of proteins from whole genomes in 2005. Nucleic Acids Res 2006;34:D169–72. 16381839 10.1093/nar/gkj148 PMC1347510

[btad473-B25] Morris JH , Knudsen GM, Verschueren E et al Affinity purification–mass spectrometry and network analysis to understand protein-protein interactions. Nat Protoc 2014;9:2539–54. 25275790 10.1038/nprot.2014.164 PMC4332878

[btad473-B26] Nepusz T , Yu H, Paccanaro A et al Detecting overlapping protein complexes in protein-protein interaction networks. Nat Methods 2012;9:471–2. 22426491 10.1038/nmeth.1938 PMC3543700

[btad473-B27] Omranian S , Angeleska A, Nikoloski Z et al PC2P: parameter-free network-based prediction of protein complexes. Bioinformatics 2021;37:73–81. 33416831 10.1093/bioinformatics/btaa1089 PMC8034538

[btad473-B28] Pan Y , Li R, Li W et al HPC-Atlas: computationally constructing a comprehensive atlas of human protein complexes. bioRxiv, 2023, preprint: not peer reviewed. 10.1016/j.gpb.2023.05.001 PMC10928439 37730114

[btad473-B29] Pu S , Wong J, Turner B et al Up-to-date catalogues of yeast protein complexes. Nucleic Acids Res 2009;37:825–31. 19095691 10.1093/nar/gkn1005 PMC2647312

[btad473-B30] Raudvere U , Kolberg L, Kuzmin I et al g:Profiler: a web server for functional enrichment analysis and conversions of gene lists (2019 update). Nucleic Acids Res 2019;47:W191–8. 31066453 10.1093/nar/gkz369 PMC6602461

[btad473-B31] Shi Y , Yao H, Guan J et al CPredictor 4.0: effectively detecting protein complexes in weighted dynamic PPI networks. IJDMB 2018;20:303–19.

[btad473-B32] Sutton RS , McAllester D, Singh S et al Policy gradient methods for reinforcement learning with function approximation. In: *Advances in Neural Information Processing Systems*, Vol. 12. 1999.

[btad473-B33] Vinyals O , Bengio S, Kudlur M. Order matters: sequence to sequence for sets. arXiv, arXiv:1511.06391, 2015, preprint: not peer reviewed.

[btad473-B34] Wang R , Wang C, Sun L et al A seed-extended algorithm for detecting protein complexes based on density and modularity with topological structure and GO annotations. BMC Genomics 2019;20:637–28. 31390979 10.1186/s12864-019-5956-y PMC6686515

[btad473-B35] Welling M , Kipf TN. Semi-supervised classification with graph convolutional networks. In: *International Conference on Learning Representations (ICLR 2017).* 2016.

[btad473-B36] Wu Z , Liao Q, Fan S et al idenPC-CAP: identify protein complexes from weighted RNA-protein heterogeneous interaction networks using co-assemble partner relation. Brief Bioinform2021a;22:bbaa372. 33333549 10.1093/bib/bbaa372

[btad473-B37] Wu Z , Liao Q, Liu B et al A comprehensive review and evaluation of computational methods for identifying protein complexes from protein–protein interaction networks. Brief Bioinform 2020;21:1531–48. 31631226 10.1093/bib/bbz085

[btad473-B38] Wu Z , Liao Q, Liu B et al idenPC-MIIP: identify protein complexes from weighted PPI networks using mutual important interacting partner relation. Brief Bioinform2021b;22:1972–83. 32065215 10.1093/bib/bbaa016

[btad473-B39] Xenarios I , Salwínski L, Duan XJ et al DIP, the database of interacting proteins: a research tool for studying cellular networks of protein interactions. Nucleic Acids Res 2002;30:303–5. 11752321 10.1093/nar/30.1.303 PMC99070

[btad473-B40] Xu B , Guan J. From function to interaction: a new paradigm for accurately predicting protein complexes based on protein-to-protein interaction networks. IEEE/ACM Trans Comput Biol Bioinform 2014;11:616–27. 26356332 10.1109/TCBB.2014.2306825

[btad473-B41] Xu K , Hu W, Leskovec J et al How powerful are graph neural networks? In: *International Conference on Learning Representations.* 2018.

[btad473-B42] Yao H , Guan J, Liu T et al Denoising protein–protein interaction network via variational graph auto-encoder for protein complex detection. J Bioinform Comput Biol2020a;18:2040010. 32698725 10.1142/S0219720020400107

[btad473-B43] Yao H , Shi Y, Guan J et al Accurately detecting protein complexes by graph embedding and combining functions with interactions. IEEE/ACM Trans Comput Biol Bioinform2020b;17:777–87. 30736004 10.1109/TCBB.2019.2897769

[btad473-B44] Yu L , Zhang W, Wang J et al seqGAN: sequence generative adversarial nets with policy gradient. In: *Proceedings of the AAAI Conference on Artificial Intelligence*, Vol. 31. 2017.

[btad473-B45] Zhang Y , Xiong Y, Ye Y et al SEAL: learning heuristics for community detection with generative adversarial networks. In: *Proceedings of the 26th ACM SIGKDD International Conference on Knowledge Discovery & Data Mining*, pp. 1103–13. 2020.

