# Supplementary material for: PCGAN: a generative approach for protein complex identification from protein interaction networks
Source: Bioinformatics. 2023 Aug 2;39(8):btad473. doi: 10.1093/bioinformatics/btad473 (PMC10457665; doi:10.1093/bioinformatics/btad473)
Supplement: btad473_Supplementary_Data [file btad473_supplementary_data.zip › Supplementary materials.docx]

**Supplementary Information**

Supplementary Table S1 Performance comparison on HuRI, BioPlex, and CPIN-H

| PPI network | | #PC | Recall | Precision | F-measure | MMR |
| --- | --- | --- | --- | --- | --- | --- |
| HuRI | 868 | | 0.170 | 0.121 | 0.141 | 0.033 |
| BioPlex | 1533 | | 0.203 | 0.154 | 0.175 | 0.047 |
| CPIN-H | **1942** | | **0.288** | **0.213** | **0.245** | **0.058** |


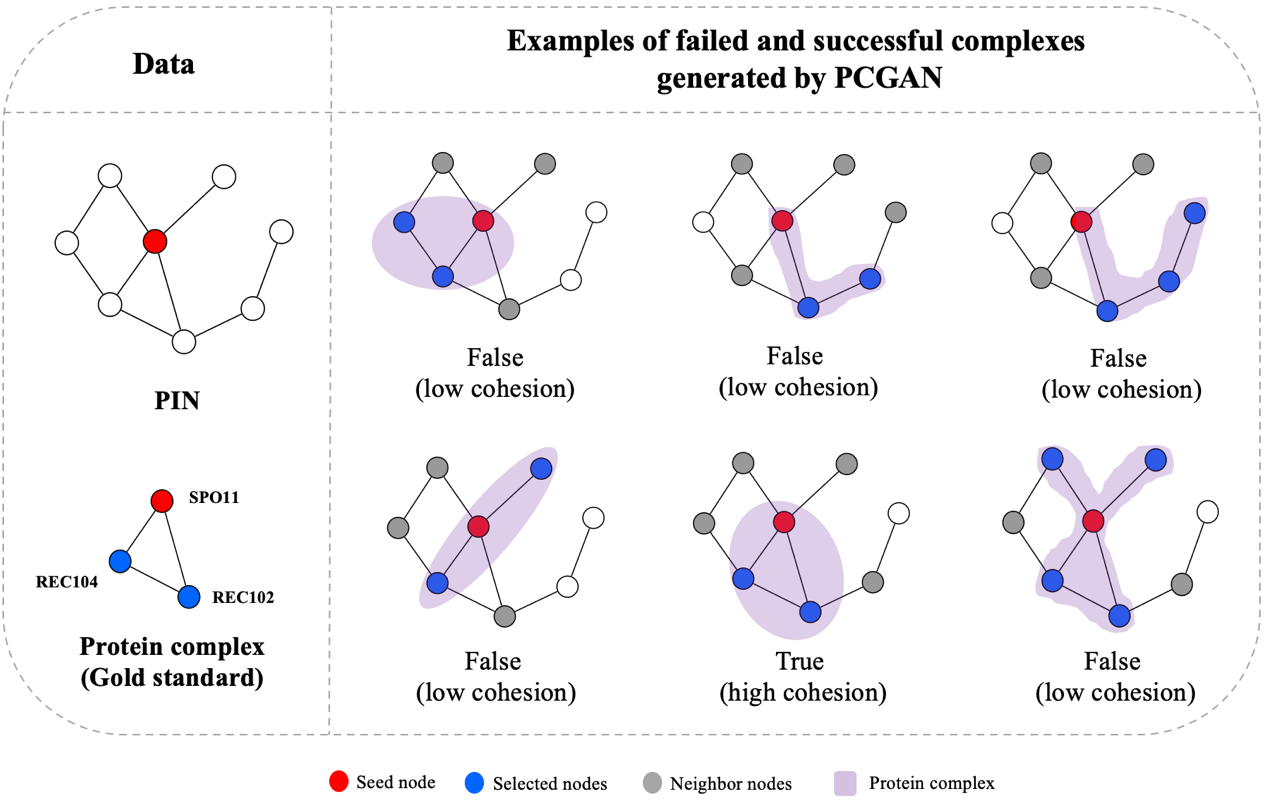


Supplementary Figure S1 Examples of failed and successful complexes generated by PCGAN with a similar seed node or protein.
