# Supplementary figures and images for: PCGAN: a generative approach for protein complex identification from protein interaction networks
Source: Bioinformatics. 2023 Aug 2;39(8):btad473. doi: 10.1093/bioinformatics/btad473 (PMC10457665; doi:10.1093/bioinformatics/btad473)

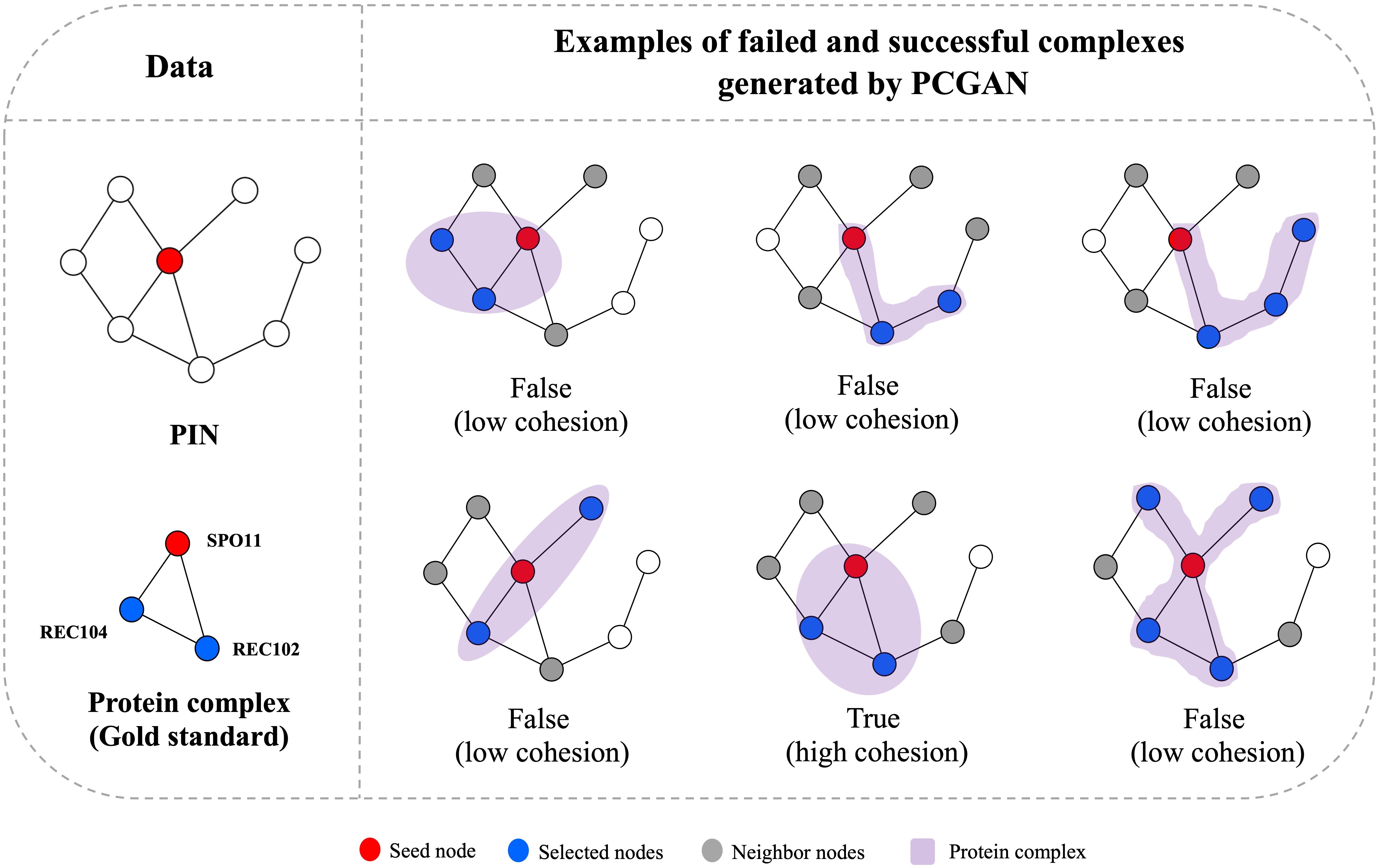

Supplement: btad473_Supplementary_Data [file btad473_supplementary_data.zip › Figure S1.jpeg]
